# Inflammatory Markers as Predictors of Diabetic Nephropathy in Type 2 Diabetes Mellitus: A Systematic Review and Meta-Analysis

**DOI:** 10.3390/medicina61020216

**Published:** 2025-01-25

**Authors:** Daniel-Corneliu Leucuța, Pauline Aurélia Fumeaux, Oana Almășan, Ștefan Lucian Popa, Abdulrahman Ismaiel

**Affiliations:** 1 Department of Medical Informatics and Biostatistics, Iuliu Hațieganu University of Medicine and Pharmacy, 400349 Cluj-Napoca, Romania; 2 Department of Prosthetic Dentistry and Dental Materials, Iuliu Hațieganu University of Medicine and Pharmacy, 32 Clinicilor Street, 400006 Cluj-Napoca, Romania; 3 2nd Department of Internal Medicine, Iuliu Hațieganu University of Medicine and Pharmacy, 400006 Cluj-Napoca, Romania

**Keywords:** diabetic nephropathy, inflammatory markers, neutrophil-to-lymphocyte ratio, platelet-to-lymphocyte ratio, systemic immune-inflammation index, diabetes mellitus type 2

## Abstract

*Background and Objectives*: Diabetic nephropathy (DN) is a major complication of diabetes mellitus and a leading cause of end-stage renal disease. Inflammatory markers such as neutrophil-to-lymphocyte ratio (NLR), platelet-to-lymphocyte ratio (PLR), systemic immune-inflammation index (SII), and red cell distribution width (RDW) have been proposed as potential predictors of DN progression. This study systematically reviews and meta-analyzes the role of these markers in DN. *Materials and Methods*: A comprehensive literature search was conducted to identify studies evaluating NLR, PLR, SII, and RDW in type 2 diabetes patients with normoalbuminuria, microalbuminuria, and macroalbuminuria. Five databases were searched: PubMed, Scopus, Embase, Web of Science, and LILACS. The Newcastle Ottawa Scale was used to assess the risk of bias in selected articles. *Results*: Out of 1556 records that were identified through searches, 40 were selected for the review. Finally, 35 were included for meta-analyses, including 13,519 patients. Higher levels of NLR, PLR, SII, and RDW were observed in macro- and microalbuminuria compared to normoalbuminuria, with significantly elevated NLR in microalbuminuria. Meta-analyses showed that NLR and RDW were significantly associated with higher odds of DN (NLR: OR 1.84, *p* < 0.001; RDW: OR 1.9, *p* = 0.023). However, PLR and SII were not significantly associated with DN. A longitudinal study confirmed SII as a significant predictor of DN progression (hazard ratio: 3.24, *p* = 0.023). *Conclusions*: This study highlights the potential of NLR and RDW as predictive markers for diabetic nephropathy.

## 1. Introduction

Diabetes is the leading cause of nephropathy, with a high mortality rate [1]. Diabetes accounts for 11.3% of fatalities worldwide [2]. The prevalence of diabetic nephropathy continues to rise, correlated with the increase in obesity and sedentary lifestyle. It affects 20% of diabetics [3]. In the United States, 54% of new cases of end-stage renal disease (ESRD) are due to diabetes. The incidence of chronic kidney disease (CKD) among diabetics is estimated at 600 patients/million/year [4].

Inflammatory markers such as the neutrophil-to-lymphocyte ratio (NLR), platelet-to-lymphocyte ratio (PLR), systemic immune-inflammation index (SII), and red cell distribution width (RDW) have emerged as potential indicators of systemic inflammation in various diseases: ovarian cancer [5], cervical cancer [6], cardiovascular disease [7], acute myocardial infarction [8], psoriasis [9], periodontitis [10], and ocular inflammation [11]. The neutrophil is a defense cell that acts as a link between innate and acquired immunity, with anti-inflammatory and anti-infectious effects. It acts rapidly through chemotaxis. Modulating its secretion of chemokines and inflammatory cytokines enables differentiation and activation of immune cells [12]. The lymphocytes play a role in both innate and acquired immunity (mainly B and T lymphocytes) [13]. Platelets, or thrombocytes, are biconvex cell fragments that play a role in primary hemostasis. They induce vascular vasoconstriction. Their levels are increased in the event of acute inflammation [14]. PLR is therefore a predictive factor for inflammatory and thrombotic states. The monocyte is a mononuclear cell of the phagocytic defense system. It plays a role in inflammation through the synthesis and secretion of cytokines, as well as in antitumor immunity and hematopoiesis [15]. SII is computed with the formula (platelet x neutrophil)/lymphocyte counts. SII reflects the degree of systemic inflammation. SII is associated with osteoporosis, cardiovascular, rheumatological, oncological, and metabolic diseases [16]. RDW is a marker of inflammation, a coefficient of variation of erythrocyte cell volume. It reflects the degree of anisocytosis [17].

We aimed, through a systematic review and meta-analysis, to evaluate the role of NLR, PLR, SII, and RDW as possible markers of diabetic nephropathy.

## 2. Materials and Methods

This systematic review was reported according to the recommendations of the “Preferred Reporting Items for Systematic Reviews and Meta-analyses Protocols (PRISMA)” [18].

### 2.1. Inclusion Criteria

In our systematic review, we included all the studies presenting patients with type 2 diabetes and evaluating the role of inflammatory markers in the development of diabetic nephropathy. The PECO strategy was based on the following: Patients (P): type 2 diabetes; Exposure (E): inflammatory markers (NLR, PLR, MLR, SII, RDW); Outcome (O): diabetic nephropathy (microalbuminuria versus normoalbuminuria or micro and macroalbuminuria versus normoalbuminuria).

### 2.2. Exclusion Criteria

We have excluded case reports, editorials, reviews, mechanistic articles, and conference abstracts from our review.

### 2.3. Search and Selection of Articles

Searching and selecting articles took place in March 2024 within the following databases: PubMed, Scopus, Embase, Web of Science, and LILACS.

### 2.4. Research Strategy

The terms used for the research of articles in the databases were “neutrophil”, “platelet”, “monocyte”, “lymphocyte”, “ratio”, “distribution of red blood cells”, “diabetes”, “renal disease”, “microalbuminuria”, “kidney” with their synonyms and abbreviations, both singular and plural variants. For PubMed, Embase, and medical subject headings, Emtree terms were also searched. The complete search strategy for all databases is presented in Supplementary Table S1.

### 2.5. Selection Process

All search results were exported in PubMed format or ris format. Then, we exported all the articles using Zotero software version 7.0.11. From there, we have created a Microsoft Excel version 2412 file. Three authors (DCL, PAF, and OA) screened the titles and summaries of the articles. Then, the full text of the initially selected articles was assessed for inclusion by three authors (DCL, PAF, and OA). Discrepancies were solved by discussion. All exclusions were documented with their reasons.

### 2.6. Data Collection Process

Data were extracted from each article using a standardized format: authors’ names and year of publication; title; publication title; country; region; study design; diabetes diagnosis criteria; nephropathy diagnosis criteria; results (NLR, PLR, LMR, SII, RDW). Three authors (OA, ȘLP, and AI) extracted the data. Discrepancies were checked against the original article and solved by discussion with (PAF and DCL).

### 2.7. Data Quality Assessment

The methodological quality of each study was rated with a modified Newcastle-Ottawa Scale (NOS). Three authors (DCL, ȘLP, and AI) assessed each article. Discrepancies were solved by discussion.

### 2.8. Assessment of Publication Bias

Publication bias was evaluated using the funnel plot and Egger test when, in the analysis, the number of studies was greater than 10.

### 2.9. Measures of Effect

Concerning the effect size, we have used the difference of means (MD) between the compared groups for quantitative biomarkers (with and without diabetic nephropathy). In the multivariate regression logistics, the odds ratio has been used as an effect size, while for the area under the receiver operating characteristic (AUC), the AUC was used.

### 2.10. Statistical Analysis

When the mean and standard deviation for the desired outcomes were unavailable, but medians were reported, we applied the formula provided by Hozo SP et al. to estimate these values from the range and sample size [19]. For studies missing specific numerical data, we retrieved the required values from their charts. Figures were digitized, and numerical data were extracted using WebPlotDigitizer version 3.1.6 [20]. For each effect size, the meta-analysis was performed with the random effects model (restricted maximum likelihood method) due to the presumption of the presence of clinical heterogeneity. Besides reporting the effect size, we reported the 95% confidence interval, *p*-value, and forest plot. The heterogeneity has been evaluated using the I^2^ and the associated *p*-value. Heterogeneity was assessed according to the Cochrane Collaboration guidelines: 0–40% was considered possibly insignificant, 30–60% indicated moderate heterogeneity, 50–90% reflected substantial heterogeneity, and 75–100% represented considerable heterogeneity [21]. Leave-one-out sensitivity analyses and subgroup analyses were performed to assess the impact of removing one article on the result and on the heterogeneity. For all statistical analyses, the R environment for statistical computing and graphics, version 4.3.2, was used [22]. The mean differences and standard errors were subjected to meta-analyses using the meta package [23].

## 3. Results

**Figure 1:**
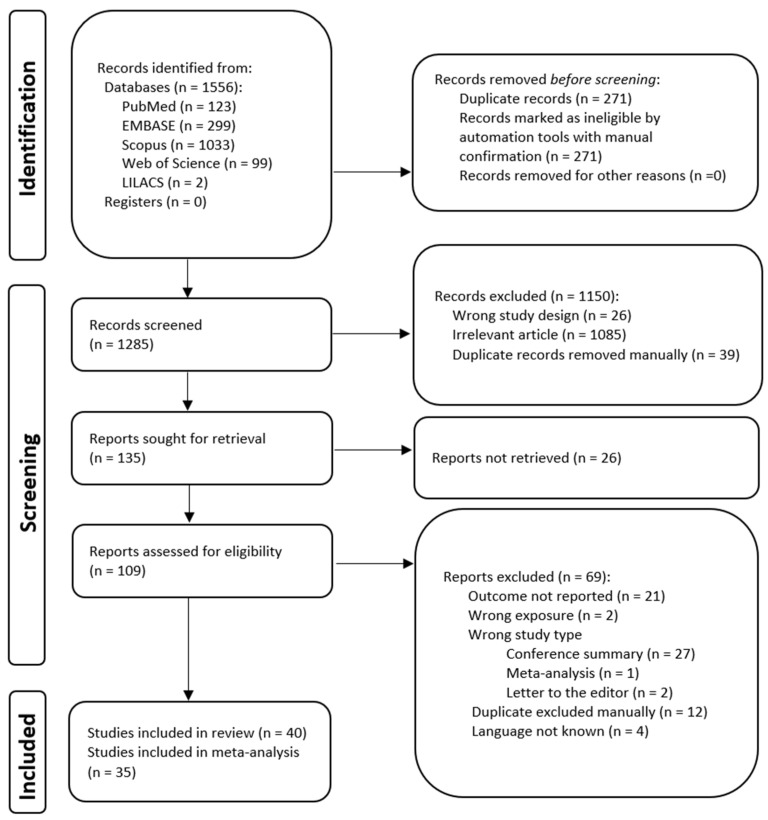
Flowchart showing the identification, screening, selection, and inclusion of articles in the systematic review.

Below is a flowchart summarizing the search and selection process (Figure 1). The search strategy was performed in March 2024. A total of 1556 records were identified through searches in databases including PubMed (n = 123), EMBASE (n = 299), Scopus (n = 1033), Web of Science (n = 99), and LILACS (n = 2). After the removal of 271 duplicate records and additional ineligible entries through automation and manual verification, 1285 records proceeded to screening. Of these, 1150 were excluded due to irrelevance (n = 1085), wrong study design (n = 26), or manual duplication (n = 39). Subsequently, 135 reports were sought for retrieval, though 26 could not be accessed. Of the 109 reports assessed for eligibility, 69 were excluded based on various criteria, including the absence of outcome data (n = 21), wrong exposure (n = 2), inappropriate study type (n = 30), duplicate study (n = 12), or unknown language (n = 4). Ultimately, 40 studies met the inclusion criteria for the review, with 35 contributing to the meta-analysis (on 13,519 patients), the most recent article being published in 2024.

### 3.1. Characteristics of the Studies

In this systematic review, we included 40 studies. Out of them, 35 were reported, three were in Africa, one was in America, and one was carried out in Europe (Table 1). Regarding the study design, 20 were cross-sectional, and eight were retrospective studies. There was a case-control study, a longitudinal study, and a comparative study. Finally, there were eight studies whose design was not reported.

Different diabetes criteria were used. Six studies were based on the definition of diabetes; five studies have used the World Health Organization criteria; five studies used the American Diabetes Association criteria; one study has referred to the Chinese Diabetes Society, another to the Chinese Diabetes Association, and 22 studies have not reported this information.

Finally, concerning stages of nephropathy, several comparisons were performed: macro, micro vs. normoalbuminuria (n = 28), micro vs. normoalbuminuria (n = 15), and macro vs. normoalbuminuria (n = 3). Some studies performed multiple types of comparisons.

### 3.2. Macro and Microalbuminuria Compared to the Group Normoalbuminuria

#### 3.2.1. Neutrophil-to-Lymphocyte Ratio

**Figure 2:**
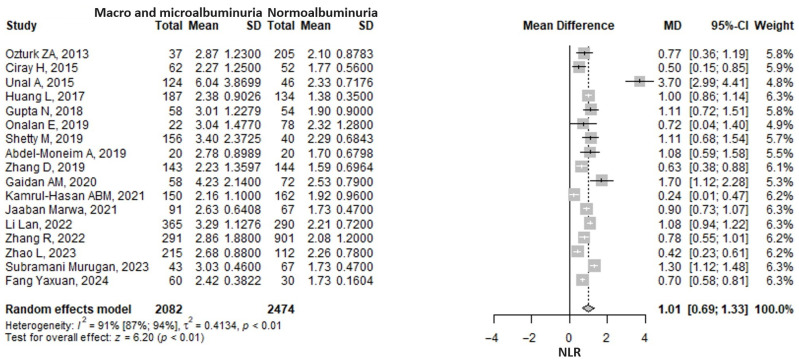
Forrest plot: the neutrophil to lymphocyte ratio (NLR) mean difference (MD) between macro- and microalbuminuria and normoalbuminuria in type 2 diabetes mellitus patients. SD, standard deviation; CI, confidence interval [24,27,28,29,35,37,40,41,42,44,48,49,54,56,58,61,62].

The meta-analysis of 17 studies showed that the neutrophil-to-lymphocyte ratio was 1.01 (95% CI: 0.69–1.33, *p* < 0.001) units higher in the macroalbuminuria/microalbuminuria group compared to the normoalbuminuria group (Figure 2). A considerable heterogeneity was found, the I2 being 90.9% (95% CI 87–93.6%), *p* ≤ 0.001. The Ünal, A., 2015 [29] study was excluded from the analysis due to its extreme values caused by the transformation of median and interquartile ranges to mean and standard deviation. The exclusion of any one study from the analysis, using a sensitivity analysis approach, did not influence the pooled result, which remained statistically significant and in the same direction (Supplementary Figure S1). The heterogeneity remained considerable, nonetheless.

#### 3.2.2. Platelet-to-Lymphocyte Ratio

**Figure 3:**
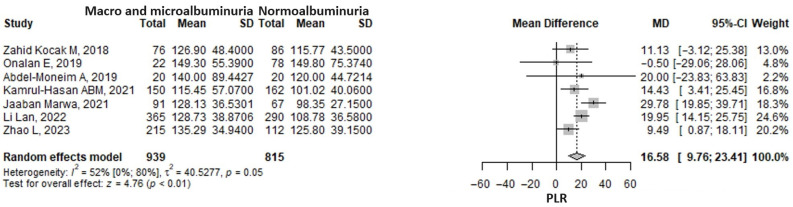
Forrest plot: the platelets to lymphocyte ratio (PLR) mean difference (MD) between macro- and microalbuminuria and normoalbuminuria in type 2 diabetes mellitus patients. SD, standard deviation; CI, confidence interval [36,37,40,48,49,54,61].

The meta-analysis of seven studies showed that the platelet-to-lymphocyte ratio (PLR) was 16.58 (95% CI: 9.76–23.41, *p* < 0.001) units higher in the macroalbuminuria/microalbuminuria group compared to the normoalbuminuria group (Figure 3). Considerable heterogeneity was found, the I2 being moderate at 51.9% (95% CI 0–79.5%), albeit borderline significant *p* = 0.052. The Tan 2023 [60] study was excluded from the analysis since it had unusually extreme values due to the transformation from median and interquartile ranges to means and standard deviations. The exclusion of any one study from the analysis, in a sensitivity analysis approach, did not influence the pooled result, which remained statistically significant and in the same direction (Supplementary Figure S2). The heterogeneity remained moderate.

#### 3.2.3. Systemic Inflammation Index

**Figure 4:**
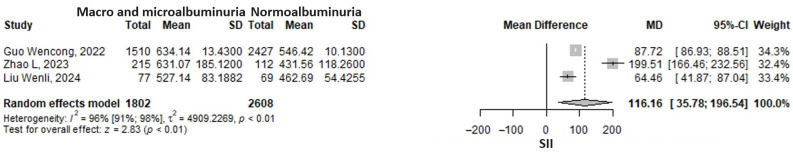
Forrest plot: the systemic inflammation index (SII) mean difference (MD) between macro- and microalbuminuria and normoalbuminuria in type 2 diabetes mellitus patients. SD, standard deviation; CI, confidence interval [52,61,63].

The meta-analysis of three studies showed that the systemic immune-inflammation index (SII) was 116.16 (95% CI: 35.78–196.54, *p* = 0.005) units higher in the macroalbuminuria/microalbuminuria group compared to the normoalbuminuria group (Figure 4). A considerable heterogeneity was found, the I2 being 95.8% (95% CI 91–98.1%), *p* ≤ 0.001. The exclusion of any one study from the analysis, in a sensitivity analysis approach, did not influence the pooled result, which remained statistically significant and in the same direction (Supplementary Figure S3), except when omitting the Guo 2022 [52] study. The heterogeneity remained considerable, nonetheless.

#### 3.2.4. Red Cell Distribution Width

**Figure 5:**
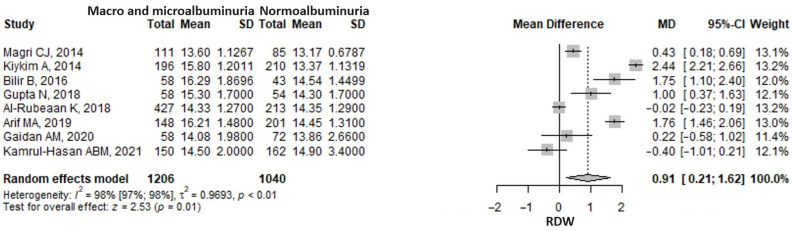
Forrest plot: the red cells distribution width (RDW) mean difference (MD) between macro- and microalbuminuria and normoalbuminuria in type 2 diabetes mellitus patients. SD, standard deviation; CI, confidence interval [25,26,31,34,35,38,44,49].

The meta-analysis of eight studies showed that the red cell distribution width (RDW) was 0.91 (95% CI: 0.21–1.62, *p* = 0.011) units higher in the macroalbuminuria/microalbuminuria group compared to the normoalbuminuria group (Figure 5). A considerable heterogeneity was found, the I2 being 97.8% (95% CI 96.9–98.4%), *p* ≤ 0.001. The exclusion of any one study from the analysis, in a sensitivity analysis approach, did not influence the pooled result, which remained statistically significant and in the same direction (Supplementary Figure S4). The heterogeneity remained considerable, nonetheless.

### 3.3. Microalbuminuria Compared to Group Normoalbuminuria

#### 3.3.1. Neutrophile to Lymphocyte Ratio

**Figure 6:**
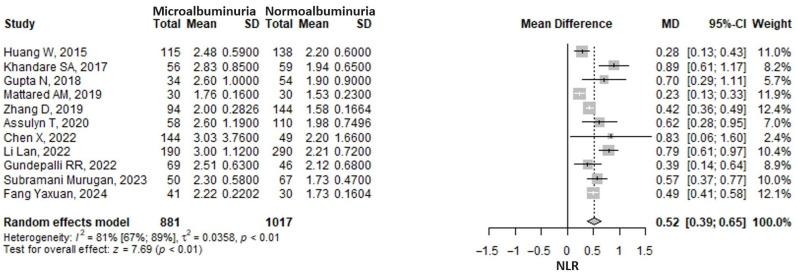
Forrest plot: the neutrophil to lymphocyte ratio (NLR) mean difference (MD) between microalbuminuria and normoalbuminuria in type 2 diabetes mellitus patients [28,33,35,39,42,43,50,51,54,58,62].

The meta-analysis showed that the neutrophil-to-lymphocyte ratio (NLR) was 0.52 (95% CI: 0.39–0.65, *p* < 0.001) units higher in the microalbuminuria group compared to the normoalbuminuria group (Figure 6). A substantial heterogeneity was found, the I2 being 81.2% (95% CI 67.4–89.2%), *p* ≤ 0.001. The exclusion of any one study from the analysis, in a sensitivity analysis approach, did not influence the pooled result, which remained statistically significant and in the same direction (Supplementary Figure S5). The heterogeneity remained considerable, nonetheless.

#### 3.3.2. Other Inflammatory Markers

The meta-analysis comparing microalbuminuria and normoalbuminuria in type 2 diabetes patients found no significant differences in PLR (MD 12.78, *p* = 0.773), SII (MD 5.48, *p* = 0.509), or RDW (MD −0.23, *p* = 0.6) (Table 2). High heterogeneity was noted for PLR (I^2^ = 99.2%) and RDW (I^2^ = 95.4%), while SII results were limited by a single study.

### 3.4. Macroalbuminuria and Normoalbuminuria

The meta-analysis found significantly higher inflammatory markers in macroalbuminuria compared to normoalbuminuria in type 2 diabetes patients (Table 3). NLR showed an MD of 1.97 (*p* < 0.001, I^2^ = 99.2%), while single-study analyses reported MDs of 77.04 for PLR and 534.58 for SII (both *p* < 0.001). High heterogeneity was noted for NLR, while PLR and SII lacked heterogeneity data.

### 3.5. Meta-Analyses of Adjusted Odds Ratios from Regressions Predicting Diabetic Nephropathy

#### 3.5.1. Neutrophil to Lymphocyte Ratio

**Figure 7:**
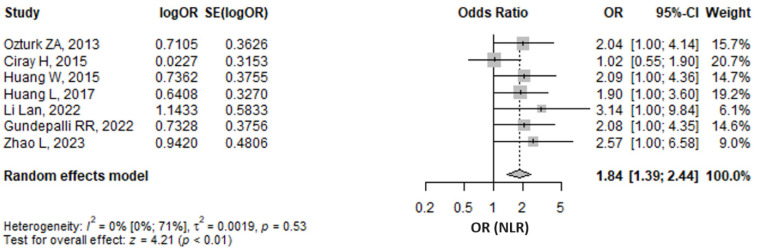
Forrest plot: the adjusted odds ratio (OR) of neutrophil to lymphocyte ratio (NLR) as a continuous variable predicting diabetic nephropathy in type 2 diabetes mellitus patients. CI, confidence interval [24,27,28,32,51,54,61].

The meta-analysis of seven studies regarding logistic regressions predicting diabetic nephropathy revealed that there were 1.84 (95% CI 1.39–2.44, *p* ≤ 0.001) higher odds for each unit increase of NLR (Figure 7). The result was found by pooling adjusted odds ratios for NLR as a continuous variable. The majority of models adjusted NLR for many confounders, as can be observed in Supplementary Table S2. The heterogeneity was not found to be important, as shown by an I^2^ of 0% (95% CI 0–70.8%), *p* = 0.533. In a sensitivity analysis approach, excluding any one study from the analysis did not influence the pool result, which remained statistically significant and in the same direction (Supplementary Figure S6).

#### 3.5.2. Platelets to Lymphocyte Ratio

**Figure 8:**
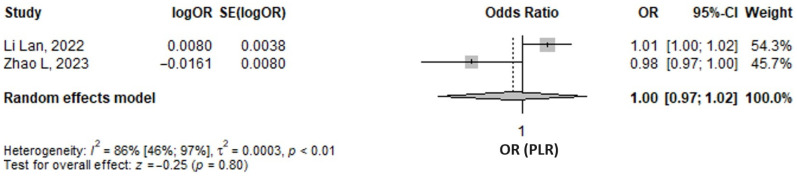
Forrest plot: the adjusted odds ratio (OR) of platelets to lymphocyte ratio (PLR) as a continuous variable predicting diabetic nephropathy in type 2 diabetes mellitus patients [54,61].

The meta-analysis of two studies regarding logistic regressions predicting diabetic nephropathy revealed that there were 1 (95% CI 0.97–1.02, *p* = 0.8) higher odds for each unit increase of PLR, as found by pooling adjusted odds ratios for PLR as a continuous variable (Figure 8). The result was not statistically significant. The majority of models adjusted PLR for many confounders, as can be observed in Supplementary Table S3. The heterogeneity was found to be substantial, as shown by an I^2^ of 86% (95% CI 46–97%), *p* < 0.001.

#### 3.5.3. Other Inflammatory Markers

The meta-analysis showed no significant association between SII and diabetic nephropathy (OR 0.51, 95% CI: −0.88–1.9, *p* = 0.469), while RDW was significantly associated with increased risk (OR 1.9, 95% CI: 0.26–3.55, *p* = 0.023) (Table 4). The majority of models for these predictors were adjusted for many confounders, as can be observed in Supplementary Tables S4 and S5. Heterogeneity could not be assessed due to the low number of studies.

#### 3.5.4. Hazard Ratios from Cox Regressions Predicting Diabetic Nephropathy Progression

Systemic Immune Inflammation Index

Only one longitudinal study (Liu, Wenly 2024 [63]) presented the hazard ratio of SII from the Cox regressions predicting diabetic nephropathy progression (Supplementary Table S6). The adjusted hazard of progression was 3.24 times higher (95% CI 1.179–8.905), *p* = 0.023.

### 3.6. The Area Under the Receiver Operating Characteristic Classifying Macro- and/or Microalbuminuria and Normoalbuminuria

#### 3.6.1. Neutrophyl to Lymphocyte Ratio

**Figure 9:**
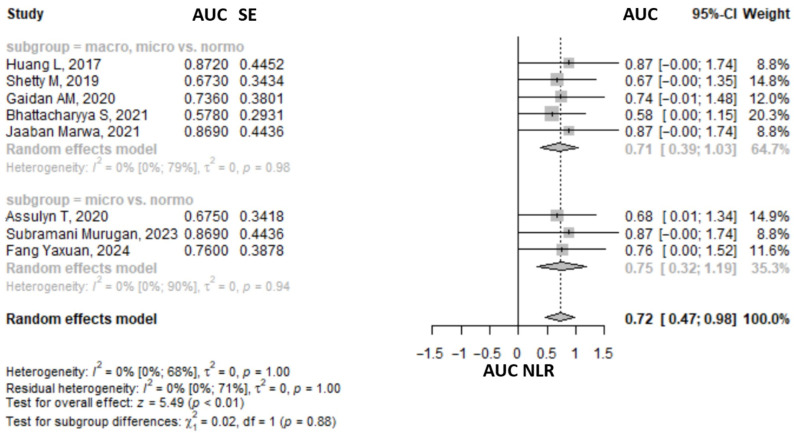
Forrest plot: the area under the receiver operator characteristic for neutrophil to lymphocyte ratio (NLR) to classify between the macro- and microalbuminuria and normoalbuminuria in type 2 diabetes mellitus patients. CI, confidence interval [32,41,43,44,47,48,58,62].

The meta-analysis of eight studies found that the AUC for NLR (Supplementary Table S7) to classify between the macro- and microalbuminuria and/or normoalbuminuria was 0.72 (95% CI: 0.47–0.98, *p* > 0.05) (Figure 9). The heterogeneity was probably not important, as suggested by the I2 of 0% (95% CI 0–67.6%), *p* = 0.999. The exclusion of any one study from the analysis, using a sensitivity analysis approach, did not influence the pooled result, which remained statistically significant and in the same direction (Supplementary Figure S7). The heterogeneity remained not important. A subgroup analysis comparing macro- and microalbuminuria vs. normoalbuminuria and microalbuminuria vs. normoalbuminuria revealed similar effect sizes (Figure 9).

#### 3.6.2. Other Inflammatory Markers

The meta-analysis showed limited predictive value for PLR (AUC 0.74, 95% CI: 0–1.48, *p* > 0.05) (Supplementary Table S8) and RDW (AUC 0.68, 95% CI: 0.2–1.16, *p* > 0.05) (Supplementary Table S9) in distinguishing albuminuria categories in type 2 diabetes patients (Table 5).

### 3.7. Publication Bias

The publication bias could be assessed for NLR, comparing macro- and microalbuminuria compared to normoalbuminuria, or for NLR, comparing microalbuminuria compared to normoalbuminuria, since there were more than ten studies included in the analyses. The funnel plots can be found in Supplementary Figures S8 and S9. No obvious asymmetry was observed on the funnel plots. The Egger test for asymmetry of the plot for the first case was *p* = 0.322, while for the second case, it was 0.158. Therefore, no clear indication of publication was found.

### 3.8. Methodological Quality Assessment of Selected Studies

The methodological quality of each study was rated using the Newcastle-Ottawa scale (Table 6).

**Figure 10:**
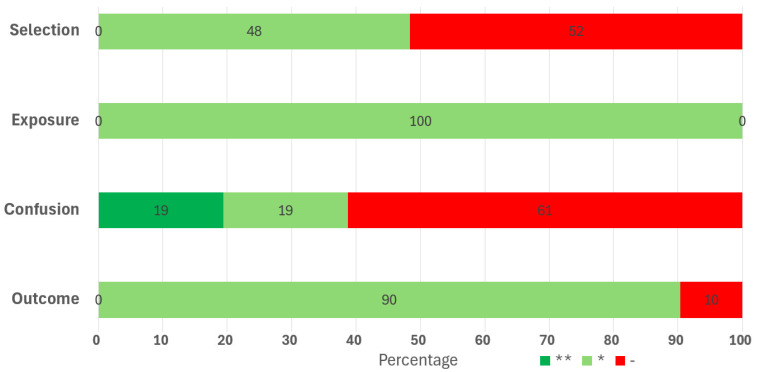
Synthesis of selected studies methodological quality with Newcastle-Ottawa Scale. *, the criteria is fulfilled; for the confusion domain, * indicates control for an important factor, while ** control for additional factors; - indicates the criteria is not fulfilled.

Figure 10 summarizes the methodological quality of all the studies. Exposure measurement (NLR, PLR, SII, RDW) was well performed in each study. With regard to the selection domain, the major inclusion criterion was type II diabetes. Only 48% explicitly reported the diagnostic criterion for this disease. However, it is very likely that even in studies that did not report the criteria, the diagnosis is trustworthy since it was performed by physicians and its criteria are commonly known. Concerning the control of confounding factors, 19% of studies used multivariate regression analyses with many adequate confounding variables, and 19% of studies with a reduced number of more or less well-chosen confounding variables. The remaining studies did not control for confounding bias. Concerning the objective, the diagnosis of diabetic nephropathy (macro- and/or microalbuminuria vs. normoalbuminuria) was well evaluated, using well-recognized classifications in 90% of studies. The remaining studies did not explicitly specify the diagnostic criteria for the objective. It is less likely that the criteria used in the remaining articles were too different from the accepted ones.

## 4. Discussion

The systematic review with meta-analysis on a large number of studies concerning the role of inflammatory markers in diabetic nephropathy offered us some relevant results. We found higher levels of NLR, PLR, SII, and RDW in macro- and microalbuminuria compared with normoalbuminuria. In addition, we found significantly higher values of NLR in microalbuminuria compared with normoalbuminuria. However, we were unable to objectify statistically significant differences between albuminuria groups concerning PLR, SII, and RDW. Then, we meta-analyzed the odds ratios of several logistic regression models predicting diabetic nephropathy with continuous markers of interest adjusted for confounders. NLR and RDW were associated with higher odds of diabetic nephropathy. A meta-analysis found the area under the curve for NLR (AUC 0.72), PLR (AUC 0.74), and RDW (AUC 0.68) in distinguishing albuminuria categories in type 2 diabetes patients, albeit non-reaching the significance threshold. We found that higher values of NLR and RDW were significantly associated with higher risks of diabetic nephropathy. In multivariate models, we found no statistically significant association between PLR, SII, and diabetic nephropathy. Moreover, one longitudinal study found that the adjusted hazard for diabetic nephropathy progression was significantly higher for increased SII values.

To support our research, we sought to compare our results with a 2018 meta-analysis by Liu, J. [64]. In this meta-analysis, only 22 studies were included on diabetic nephropathy. Among them, 12 studies on 2404 patients with diabetic nephropathy and five articles on the level of albuminuria (micro- and macroalbuminuria) investigated NLR. This meta-analysis concluded that NLR was significantly higher in patients with diabetic nephropathy or macroalbuminuria than in those without diabetic nephropathy or with microalbuminuria. This result is consistent with our own findings of higher NLR levels in diabetic nephropathy or micro/macroalbuminuria than in normoalbuminuria. This study also looked at other indicators that were not of interest to our study—mean thrombocyte volume and thrombocyte distribution width—for which they observed greater mean values for cases of diabetic nephropathy or micro/macroalbuminuria compared with normoalbuminuria. To the best of our knowledge, there are no other systematic reports with meta-analyses on the same subject as our study.

Changes in hemodynamics and metabolism linked to diabetes mellitus (DM) cause several transduction pathways in almost all types of kidney cells to become activated [65]. Diabetes-related metabolic and hemodynamic problems, such as hyperglycemia and advanced glycation end products, trigger the mononuclear phagocyte system resident in the kidney, which in turn triggers the production of paracrine signals and proinflammatory cytokines [66,67]. Important molecules, pathways, the nuclear transcription factor kappa B, the Janus kinase/signal transducers and activators of transcription (JAK/STAT) pathway, and inflammatory cytokines (interleukins such as IL-6, IL-18, and tumor necrosis factor-alpha—TNF-α), Intercellular Adhesion Molecule 1, are implicated in both systemic and local renal inflammation in DKD [68,69]. Macrophages are the most common cell type that is recruited by the kidney [67,70]. Cytokines generated by resident macrophages and other kidney cells attract more monocytes and macrophages [71]. Inflammatory-related structural alterations linked to DKD are the final result of a cycle of cytokine release and monocyte and macrophage recruitment [71,72]. Macrophages, in their turn, recruit neutrophils and lymphocytes. The recruitment of neutrophils and lymphocytes can modify the hematological ratios, such as NLR, PLR, and SII. Diabetes induces platelet activation [73]. Moreover, besides their role in hemostasis and thrombosis, platelets are actively implicated in inflammation modulation [74]. This contributes to the chronic kidney disease pathophysiology [74]. Platelets in their turn interact with other blood cells, like neutrophils [75]. Thus, PLR and SII hematological ratios could be modified during the kidney disease pathophysiology.

### 4.1. Study Limitations

Our study has a number of limitations. For several indicators, the statistical heterogeneity of the results was statistically significant and important. We also observed clinical heterogeneity in terms of subject characteristics. To deal with this situation, we used a random-effects model and leave-one-out sensitivity analyses. The results were robust, remaining statistically significant and going in the same direction as the original results after the sensitivity analyses. A possible classification bias might have been introduced by the diagnostic criteria for diabetes and diabetic nephropathy. The univariate analyses comparing those with diabetic nephropathy and those without nephropathy concerning continuous biomarkers are not protected from confounding. This is why we extracted the results of multivariate logistic regression predicting diabetic nephropathy. Here, many confounders were taken into account in the selected studies. Nevertheless, since the included studies are observational, residual confounding cannot be ruled out.

### 4.2. Study Strengths

Our study has several strong points. Firstly, the search strategy was carried out in a large number of bibliographic databases—five. The search strategy was complex, using the medical subject heading terms MeSH or Emtree (for Embase), as well as words searched in all fields of these two databases, with singular and plural variants, synonyms, and abbreviations. Secondly, the meta-analysis was carried out according to the availability of data on either the macro- and microalbuminuria group or the microalbuminuria group, compared with the reference group—with normoalbuminuria—to avoid any loss of information and to improve its accuracy. Thirdly, the meta-analysis was initially carried out using univariate analyses, comparing biomarker levels between the two groups. The analyses were then performed on multivariate logistic regressions, taking into account confounding factors. Compared with the systematic report by Liu, J. from 2018 [64] published on a topic similar to ours, our study was carried out on a larger number of databases, and the search strategy was more complex. Our study also identified many more studies on the subject (almost double) and additionally performed meta-analysis on multivariate regressions. Finally, it identified more markers of inflammation in addition to NLR, PLR, and SII compared to the other meta-analysis.

### 4.3. Clinical Utility

From a clinical standpoint, our findings imply that early detection and risk classification of individuals at increased risk for diabetic nephropathy (DN) may be aided by the monitoring and possible optimization of inflammatory markers like NLR and RDW, and, to a lesser extent, PLR and SII. Although these markers cannot be changed like more conventional risk factors like blood pressure or blood sugar, addressing the underlying inflammatory processes that are represented by elevated NLR and RDW, by means of tactics like managing comorbidities, improving glycemic control, and investigating anti-inflammatory therapies, may lower the chance of developing or worsening DN and its complications. Incorporating these markers into routine clinical evaluations might improve the early diagnosis and treatment of high-risk individuals, but further interventional research is required to clarify whether addressing inflammation can directly alter DN outcomes.

## 5. Conclusions

This systematic review and meta-analysis of a large number of studies highlight the potential of inflammatory markers in diabetic nephropathy. Higher NLR, PLR, SII, and RDW levels were observed in macro- and microalbuminuria compared to normoalbuminuria, with NLR significantly elevated in microalbuminuria. NLR and RDW were associated with higher odds of diabetic nephropathy in the pooled analysis of results from multivariate logistic regressions. Moreover, one longitudinal study found that the adjusted hazard for diabetic nephropathy progression was significantly higher for increased SII values.

## Figures and Tables

**Table 1:** Study characteristics.

Publication Year	Study Name	Country	Region	Study Design (Prospective/Retrospective/Cross-Sectional)	Diabetes Criteria, Detailed	Diabetic Nephropathy Stage
2013	Ozturk, 2013 [24]	Turkey	Asia	cross-sectional	Specified	macro, micro vs. normo
2014	Kiykim, 2014 [25]	Turkey	Asia	retrospective	Specified	macro, micro vs. normo
2014	Magri, 2014 [26]	Malta	Europe	cross-sectional	NR	macro, micro vs. normo
2015	Ciray, 2015 [27]	Turkey	Asia	cross-sectional	NR	macro, micro vs. normo
2015	Huang, 2015 [28]	China	Asia	NR	NR	micro vs. normo
2015	Ünal, 2015 [29]	Turkey	Asia	NR	NR	macro, micro vs. normo
2015	Zhang, 2015 [30]	China	Asia	cross-sectional	Specified	micro vs. normo
2016	Bilir, 2016 [31]	Turkey	Asia	retrospective	NR	macro, micro vs. normo
2017	Huang, 2017 [32]	China	Asia	NR	WHO	macro, micro vs. normo
2017	Khandare, 2017 [33]	India	Asia	cross-sectional	NR	micro vs. normo
2018	Al-Rubeaan, 2018 [34]	Saudi Arabia	Asia	cross-sectional	ADA 2005	macro, micro vs. normo
2018	Gupta, 2018 [35]	Turkey	Asia	NR	NR	microphone vs. normal; macro, micro vs. normal
2018	Zahid Kocak, 2018 [36]	Turkey	Asia	NR	NR	macro, micro vs. normo
2019	Abdel-Moneim, 2019 [37]	Egypt	Africa	NR	NR	macro, micro vs. normo
2019	Arif, 2019 [38]	Pakistan	Asia	cross-sectional	NR	macro, micro vs. normo
2019	Mattared, 2019 [39]	Egypt	Africa	NR	ADA 2017	micro vs. normo
2019	Onalan, 2019 [40]	Turkey	Asia	NR	NR	macro, micro vs. normo
2019	Shetty, 2019 [41]	India	Asia	cross-sectional	NR	macro, micro vs. normo
2019	Zhang, 2019 [42]	China	Asia	cross-sectional	WHO 1999	microphone vs. normal; macro, micro vs. normal
2020	Assulyn, 2020 [43]	Israel	Asia	retrospective	NR	micro vs. normo
2020	Gaidan, 2020 [44]	Iraq	Asia	cross-sectional	ADA	macro, micro vs. normo
2020	Huang, 2020 [45]	China	Asia	NR	CDA 2013	macro, micro vs. normo
2020	Kocak, 2020 [46]	Turkey	Asia	retrospective	NR	micro vs. normo
2021	Bhattacharyya, 2021 [47]	India	Asia	cross-sectional/prospective	Specified	macro, micro vs. normo
2021	Jaaban, 2021 [48]	Syria	Asia	NR	NR	macro, micro vs. normo
2021	Kamrul-Hasan, 2021 [49]	Bangladesh	Asia	cross-sectional	Specified	macro, micro vs. normo
2022	Chen, 2022 [50]	China	Asia	retrospective	NR	micro vs. normo
2022	Gundepalli, 2022 [51]	India	Asia	case control	NR	micro vs. normo
2022	Guo, 2022 [52]	USA	America	nationally representative cross-sectional survey	Specified	macro, micro vs. normo
2022	Gurmu, 2022 [53]	Ethiopia	Africa	comparative	NR	macro vs. normo
2022	Li, 2022 [54]	China	Asia	cross-sectional study	WHO	microphone vs. normal; macro, micro vs. normal
2022	Singh, 2022 [55]	India	Asia	cross-sectional	ADA 2019	macro vs. normo
2022	Zhang, 2022 [56]	China	Asia	cross-sectional	CDS 2019	macro, micro vs. normo
2023	Moh, 2023 [57]	Singapore	Asia	longitudinal study	ADA	macro, micro vs. normo
2023	Subramani, 2023 [58]	India	Asia	cross sectional	NR	microphone vs. normal; macro, micro vs. normal
2023	Suvarna, 2023 [59]	India	Asia	retrospective study	NR	macro vs. normo
2023	Tan, 2023 [60]	China	Asia	cross-sectional	NR	micro vs. normo
2023	Zhao, 2023 [61]	China	Asia	retrospective study	WHO 1999	macro, micro vs. normo
2024	Fang, 2024 [62]	China	Asia	cross-sectional	NR	microphone vs. normal; macro, micro vs. normal
2024	Liu, 2024 [63]	China	Asia	retrospective non-interventional study	WHO	microphone vs. normal; macro, micro vs. normal

ADA, American Diabetes Association; WHO, World Health Organization; CDA, Chinese Diabetes Association; CDS, Chinese Diabetes Society; NR, not reported; Specified, specific definition of diabetes.

**Table 2:** Meta-analyses results between microalbuminuria and normoalbuminuria in type 2 diabetes mellitus patients concerning PLR, SII, and RDW, presenting mean differences and heterogeneity.

Characteristic	N Studies	N Micro Albuminuria	N Normo Albuminuria	MD (95% CI)	*p*-Value	I^2^ (95% CI)
PLR	3	389	394	12.78 (−74.13–99.69)	0.773	99.2 (98.8–99.5)
SII	1	69	157	5.48 (−10.78 –21.74)	0.509	NC
RDW	3	210	366	−0.23 (−1.1–0.63)	0.6	95.4 (89.8–97.9)

N, number of participants; MD, mean difference; CI, confidence interval; PLR, trays to lymphocyte ratio; SII, systemic inflammation index; RDW, red cell distribution width; NC, cannot be computed due to the small number of studies.

**Table 3:** Meta-analyses results between macroalbuminuria and normoalbuminuria in type 2 diabetes mellitus patients concerning NLR, PLR, and SII presenting mean differences, heterogeneity, and publication bias.

Characteristic	N Studies	N Micro Albuminuria	N Normo Albuminuria	MD (95% CI)	*p*-Value	I^2^ (95% CI)	Characteristic
NLR	3	291	432	1.97 (0.95–2.99)	<0.001	99.2 (98.7–99.5)	<0.001
PLR	1	100	100	77.04 (70.94–83.15)	<0.001	NC	
SII	1	100	100	534.58 (485.4 5–583.7)	<0.001	NC	

N, number of participants; MD, mean difference; CI, confidence interval; PLR, trays to lymphocyte ratio; SII, systemic inflammation index; NC, cannot be computed due to the small number of studies.

**Table 4:** Meta-analyses results in type 2 diabetes mellitus patients concerning SII and RDW as continuous variables predicting diabetic nephropathy presenting odds ratios.

Characteristic, Effect Size Type	N Studies	OR (95% CI)	*p*-Value	Studies
SII	2	0.51 (−0.88–1.9)	0.469	Zhao L, 2023 [61]; Liu Wenli, 2024 [63]
RDW	2	1.9 (0.26–3.55)	0.023	Magri CJ, 2014 [26]; Zhang M, 2015 [30]

N, number of participants; CI, confidence interval; SII, systemic inflammation index; RDW, red cells distribution width.

**Table 5:** Meta-analyses result in type 2 diabetes mellitus patients concerning the area under the receiver operator characteristic for PLR and RDW to classify between the macro- and microalbuminuria and/or normoalbuminuria.

Characteristic, Effect Size Type	N Studies	AUC (95% CI)	*p*-Value	Studies
PLR	1	0.74 (0–1.48)	0.526	Jaaban Marwa, 2021 [48]
RDW	2	0.68 (0.2–1.16)	0.466	Zhang M, 2015 [30]; Assulyn T, 2020 [43]

N, number; AUC, area under the receiver operator characteristic; CI, confidence interval; PLR, trays to lymphocyte ratio; RDW, red cell distribution width.

**Table 6:** The methodological quality of selected studies was assessed with Newcastle-Ottawa Scale.

Kind of Study	No Study	Selection	Exposure	Confusion	Objective
case-control	Tan 2023 [60]	-	*	-	-
case-control	Gundepalli 2022 [51]	-	*	**	*
cross-sectional	Bhattacharyya 2021 [47]	*	*	-	*
cross-sectional	Chen, 2022 [50]	-	*	-	*
cross-sectional	Arif 2019 [38]	-	*	-	*
cross-sectional	Moh 2023 [57]	*	*	-	*
cross-sectional	Zhang 2015 [30]	*	*	*	*
cross-sectional	Li, 2022 [54]	*	*	-	*
cross-sectional	Zhao 2023 [61]	*	*	**	*
cross-sectional	Shetty 2019 [41]	-	*	-	*
cross-sectional	Abdel- Moneim 2019 [37]	-	*	-	-
cross-sectional	Kamrul-Hasan 2021 [49]	*	*	-	*
cross-sectional	Bilir 2016 [31]	-	*	-	*
cross-sectional	Fang 2024 [62]	-	*	*	*
cross-sectional	Zhang 2022 [56]	*	*	-	*
cross-sectional	Ozturk 2013 [24]	*	*	*	*
cross-sectional	Mattared 2019 [39]	*	*	-	*
cross-sectional	Zahid Kocak 2018 [36]	-	*	-	-
cross-sectional	Kocak 2020 [46]	-	*	**	*
cross-sectional	Huang, 2020 [45]	*	*	**	*
cross-sectional	Ciray 2015 [27]	-	*	*	*
cross-sectional	Singh 2022 [55]	*	*	-	*
cross-sectional	Jaaban 2021 [48]	-	*	-	*
cross-sectional	Gurmu 2022 [53]	-	*	-	*
cross-sectional	Magri 2014 [26]	-	*	*	*
cross-sectional	Gaidan 2020 [44]	*	*	**	*
cross-sectional	Subramani 2023 [58]	-	*	-	*
cross-sectional	Khandare 2017 [33]	-	*	-	*
cross-sectional	Guo 2022 [52]	*	*	**	*
cross-sectional	Zhang 2019 [42]	*	*	-	*
cross-sectional	Al-Rubeaan 2018 [34]	*	*	*	*

*, the criteria is fulfilled; for the confusion domain, * indicates control for an important factor, while ** control for additional factors; - indicates the criteria is not fulfilled.

## Data Availability

Data are contained within the article.

## Supplementary Materials

The following supporting information can be downloaded at: https://www.mdpi.com/article/10.3390/medicina61020216/s1, Figure S1: Leave-one-out sensitivity analysis plot for selected studies for NLR mean difference between normoalbuminuria and macroalbuminuria + microalbuminuria; Figure S2: Leave-one-out sensitivity analysis plot for selected studies for PLR mean difference between normoalbuminuria and macroalbuminuria + microalbuminuria; Figure S3: Leave-one-out sensitivity analysis plot for selected studies for SII mean difference between normoalbuminuria and macroalbuminuria + microalbuminuria; Figure S4: Leave-one-out sensitivity analysis plot for selected studies for RDW mean difference between normoalbuminuria and macroalbuminuria + microalbuminuria. Figure S5: Leave-one-out sensitivity analysis plot for selected studies for NLR mean difference between normoalbuminuria and microalbuminuria; Figure S6: Leave-one-out sensitivity analysis plot for selected studies for NLR odds ratio for predicting diabetic nephropathy; Figure S7: Leave-one-out sensitivity analysis plot for selected studies for AUC of NLR classifying between diabetic nephropathy and normoalbuminuria; Figure S8: Funnel plot for the mean difference in neutrophil to lymphocyte ratio (NLR) between macro- and microalbuminuria and normoalbuminuria for patients with type 2 diabetes; Figure S9: Funnel plot for the mean difference in neutrophil to lymphocyte ratio (NLR) between microalbuminuria and normoalbuminuria for patients with type 2 diabetes; Table S1: Search strategies in multiple databases; Table S2: Logistic regression results predicting diabetic nephropathy for neutrophil to lymphocyte ratio study results; Table S3: Logistic regression results predicting diabetic nephropathy for platelets to lymphocyte ratio study results; Table S4: Logistic regression results predicting diabetic nephropathy for systemic inflammation index study results; Table S5: Logistic regression results predicting diabetic nephropathy for red cell distribution width study results; Table S6: Cox regression results predicting diabetic nephropathy for SII study results; Table S7: Area under the curve for neutrophil to lymphocyte ratio study results; Table S8: Area under the curve for platelets to lymphocyte ratio study results; Table S9: Area under the curve for red cell distribution width study results.

## Abbreviations

The following abbreviations are used in this manuscript:

ESRD	End-stage renal disease
CKD	Chronic kidney disease
NLR	Neutrophil-to-lymphocyte ratio
PLR	Platelet-to-lymphocyte ratio
SII	Systemic immune-inflammation index
RDW	Red cell distribution width
PRISMA	Preferred Reporting Items for Systematic Reviews and Meta-analyses
ADA	American Diabetes Association
WHO	World Health Organization
CDA	Chinese Diabetes Association
CDS	Chinese Diabetes Society
MD	Mean difference
AUC	Area under the curve
OR	Odds ratio
CI	Confidence interval
SD	Standard deviation
NOS	Newcastle-Ottawa Scale
