# Supplementary material for: Inflammatory Markers as Predictors of Diabetic Nephropathy in Type 2 Diabetes Mellitus: A Systematic Review and Meta-Analysis
Source: Medicina (Kaunas). 2025 Jan 25;61(2):216. doi: 10.3390/medicina61020216 (PMC11857583; doi:10.3390/medicina61020216)
Supplement: Supplementary file 1 [file medicina-61-00216-s001.zip › medicina-3417359-supplementary.pdf]

Contents

Search strategies 1

Sensitivity analyses 4

NLR mean difference between normoalbuminuria and macroalbuminuria + microalbuminuria 4

PLR mean difference between normoalbuminuria and macroalbuminuria + microalbuminuria 5

SII mean difference between normoalbuminuria and macroalbuminuria + microalbuminuria 6

RDW mean difference between normoalbuminuria and macroalbuminuria + microalbuminuria 7

NLR mean difference between normoalbuminuria and microalbuminuria 8

NLR odds ratio for predicting diabetic nephropathy 9

NLR for AUC of NLR classifying between diabetic nephropathy and normoalbuminuria 10

Publication bias analyses 11

Regression results predicting diabetic nepthropathy 13

Area under the curve study results 17

Area under the curve for NLR study results 17

Area under the curve for PLR study results 18

Search strategies

Suplementary Table S1. Search strategies in multiple databases.

Pubmed
(((((("neutrophils"[MeSH Terms] OR "neutrophil"[All Fields] OR "neutrophile"[All Fields] OR "neutrophiles"[All Fields] OR "neutrophils"[All Fields] OR "Polymorphonuclear"[All Fields] OR "granulocyte"[All Fields] OR "pmn"[TIAB] OR "pmn"[OT]) OR ("Blood Platelets"[Mesh] OR "Platelet Count"[Mesh] OR "platelet"[All Fields] OR "platelets"[All Fields] OR "Thrombocyte"[All Fields] OR "Thrombocytes"[All Fields]) OR ("Monocytes"[Mesh] OR "Monocyte"[All Fields] OR "Monocytes"[All Fields])) AND ("lymphocytes"[MeSH Terms] OR "lymphocyte count"[MeSH Terms] OR "lymphocyte"[All Fields] OR "lymphocytes"[All Fields] OR "lymphocyts"[All Fields]) AND ("ratio"[All Fields] OR "ratios"[All Fields])) OR "PLR"[All Fields] OR "NLR"[All Fields] OR "LMR"[All Fields] OR "Systemic immune-inflammation index"[All Fields] OR "SII"[All Fields]

)OR ("red cell distribution width"[All Fields] OR RDW[Title/Abstract])) AND ("diabetes mellitus, type 2"[MeSH Terms] OR ("diabetes"[All Fields] AND ("non insulin dependent"[All Fields] OR "non-insulin dependent"[All Fields] OR "type 2"[All Fields] OR "type II"[All Fields] OR "T2DM"[TIAB]))) AND (("diabetic nephropathies"[MeSH Terms] OR ("diabetic"[All Fields] AND ("nephropathy"[All Fields] OR "nephropathies"[All Fields] OR "glomerulopathy"[All Fields] OR "glomerulosclerosis"[All Fields]))) OR ("kidney diseases"[MeSH Terms] OR ("kidney"[All Fields] AND "disease"[All Fields]) OR ("renal"[All Fields] AND "disease"[All Fields]) OR "kidney disease"[All Fields] OR ("renal"[All Fields] AND "diseases"[All Fields]) OR ("kidney"[All Fields] AND "diseases"[All Fields]) OR "kidney diseases"[All Fields]) OR microalbuminuria[All Fields] OR ("kidney"[MeSH Terms] OR "kidney"[All Fields] OR "kidneys"[All Fields] OR "renal"[All Fields]))) NOT ("nlrp"[All Fields] OR "nlrp3"[All Fields])
EMBASE
((((((('neutrophil'/exp OR 'neutrophil count'/exp OR 'neutrophil' OR 'neutrophile' OR 'neutrophiles' OR 'neutrophils' OR 'Polymorphonuclear' OR 'granulocyte' OR 'pmn' OR 'pmn') OR ('thrombocyte'/exp 'Blood Platelets' OR 'Platelet Count'/exp OR 'platelet' OR 'platelets' OR 'Thrombocyte' OR 'Thrombocytes') OR ('monocyte'/exp OR 'monocyte count'/exp OR 'monocyte' OR 'monocytes')) AND ('lymphocyte'/exp OR 'lymphocyte count'/exp OR 'lymphocyte' OR 'lymphocytes' OR 'lymphocyts') AND ('ratio' OR 'ratios')) OR 'PLR' OR 'NLR' OR 'LMR' OR 'neutrophil lymphocyte ratio'/exp OR 'platelet lymphocyte ratio'/exp OR 'monocyte lymphocyte ratio'/exp OR 'systemic immune inflammation index'/exp OR 'Systemic immune-inflammation index' OR 'SII') OR ('red cell distribution width'/exp OR 'red cell distribution width' OR RDW)) AND ('non insulin dependent diabetes mellitus'/exp OR ('diabetes' AND ('non insulin dependent' OR 'non-insulin dependent' OR 'type 2' OR 'type II' OR 'T2DM'))) AND (('diabetic nephropathy'/exp OR ('diabetic' AND ('nephropathy' OR 'nephropathies' OR 'glomerulopathy' OR 'glomerulosclerosis'))) OR ('kidney diseases'/exp OR (('kidney' OR 'renal') AND ('disease' OR 'diseases'))) OR microalbuminuria OR ('kidney'/exp OR 'kidney' OR 'kidneys' OR 'renal'))) NOT ('nlrp' OR 'nlrp3')
Scopus
ALL (((((((neutrophil OR neutrophile OR neutrophiles OR neutrophils OR polymorphonuclear OR granulocyte OR pmn) OR (blood AND platelets OR platelet OR platelets OR thrombocyte OR thrombocytes) OR (monocyte OR monocytes)) AND (lymphocyte OR lymphocytes OR lymphocyts) AND (ratio OR ratios)) OR plr OR nlr OR lmr OR "neutrophil lymphocyte ratio" OR "platelet lymphocyte ratio" OR "monocyte lymphocyte ratio" OR "systemic immune inflammation index" OR "Systemic immune-inflammation index" OR sii) OR ("red cell distribution width" OR rdw))

AND ("diabetes" AND ("non insulin dependent" OR "non-insulin dependent" OR "type 2" OR "type II" OR "T2DM"))<br>AND ("diabetic" AND ("nephropathy" OR "nephropathies" OR "glomerulopathy" OR "glomerulosclerosis"))) AND NOT (nlrp OR nlrp3)) AND (LIMIT-TO (DOCTYPE , "ar")) AND (LIMIT-TO (SUBJAREA , "MEDI")) AND (LIMIT-TO (SRCTYPE , "j"))
Web of science
TS=(((((((neutrophil OR neutrophile OR neutrophiles OR neutrophils OR polymorphonuclear OR granulocyte OR pmn) OR (blood AND platelets OR platelet OR platelets OR thrombocyte OR thrombocytes) OR (monocyte OR monocytes)) AND (lymphocyte OR lymphocytes OR lymphocyts) AND (ratio OR ratios)) OR plr OR nlr OR lmr OR "neutrophil lymphocyte ratio" OR "platelet lymphocyte ratio" OR "monocyte lymphocyte ratio" OR "systemic immune inflammation index" OR "Systemic immune-inflammation index" OR sii) OR ("red cell distribution width" OR rdw))<br>AND ("diabetes" AND ("non insulin dependent" OR "non-insulin dependent" OR "type 2" OR "type II" OR "T2DM")) AND (("diabetic" AND ("nephropathy" OR "nephropathies" OR "glomerulopathy" OR "glomerulosclerosis"))OR (("kidney" OR "renal") AND ("disease" OR "diseases"))OR microalbuminuria OR (kidney OR kidneys OR renal))) NOT ("nlrp" OR "nlrp3"))
LILACS
tw:(((((((neutrophil OR neutrophile OR neutrophiles OR neutrophils OR polymorphonuclear OR granulocyte OR pmn) OR (blood AND platelets OR platelet OR platelets OR thrombocyte OR thrombocytes) OR (monocyte OR monocytes)) AND (lymphocyte OR lymphocytes OR lymphocyts) AND (ratio OR ratios)) OR plr OR nlr OR lmr OR "neutrophil lymphocyte ratio" OR "platelet lymphocyte ratio" OR "monocyte lymphocyte ratio" OR "systemic immune inflammation index" OR "Systemic immune-inflammation index" OR sii) OR ("red cell distribution width" OR rdw))<br>AND ("diabetes" AND ("non insulin dependent" OR "non-insulin dependent" OR "type 2" OR "type II" OR "T2DM")) AND<br>(("diabetic" AND ("nephropathy" OR "nephropathies" OR "glomerulopathy" OR "glomerulosclerosis")) OR<br>(("kidney" OR "renal") AND ("disease" OR "diseases"))OR microalbuminuria OR (kidney OR kidneys OR renal))) NOT ("nlrp" OR "nlrp3"))

Sensitivity analyses

NLR mean difference between normoalbuminuria and macroalbuminuria + microalbuminuria

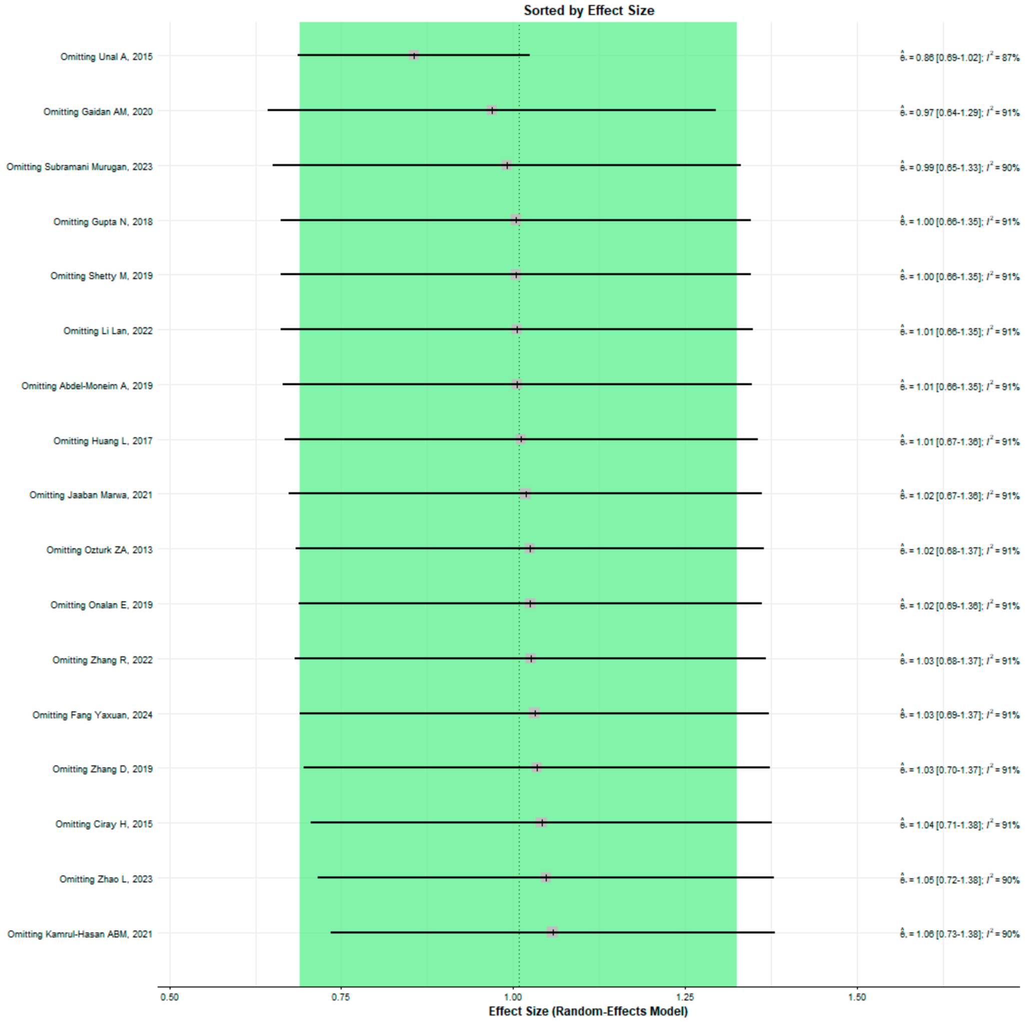

Supplementary Figure S1. Leave-one-out sensitivity analysis plot for selected studies for NLR mean difference between normoalbuminuria and macroalbuminuria + microalbuminuria.

PLR mean difference between normoalbuminuria and macroalbuminuria + microalbuminuria

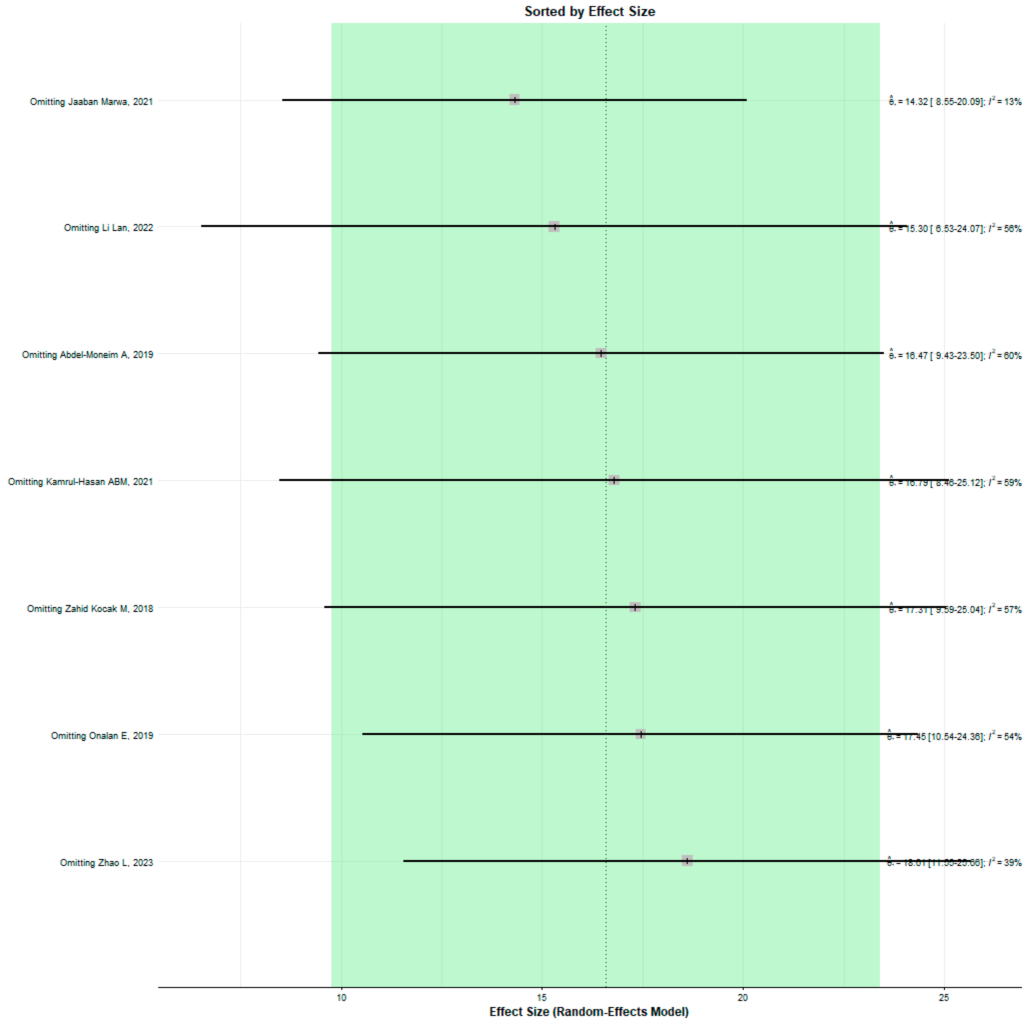

Supplementary Figure S2. Leave-one-out sensitivity analysis plot for selected studies for PLR mean difference between normoalbuminuria and macroalbuminuria + microalbuminuria.

SII mean difference between normoalbuminuria and macroalbuminuria + microalbuminuria

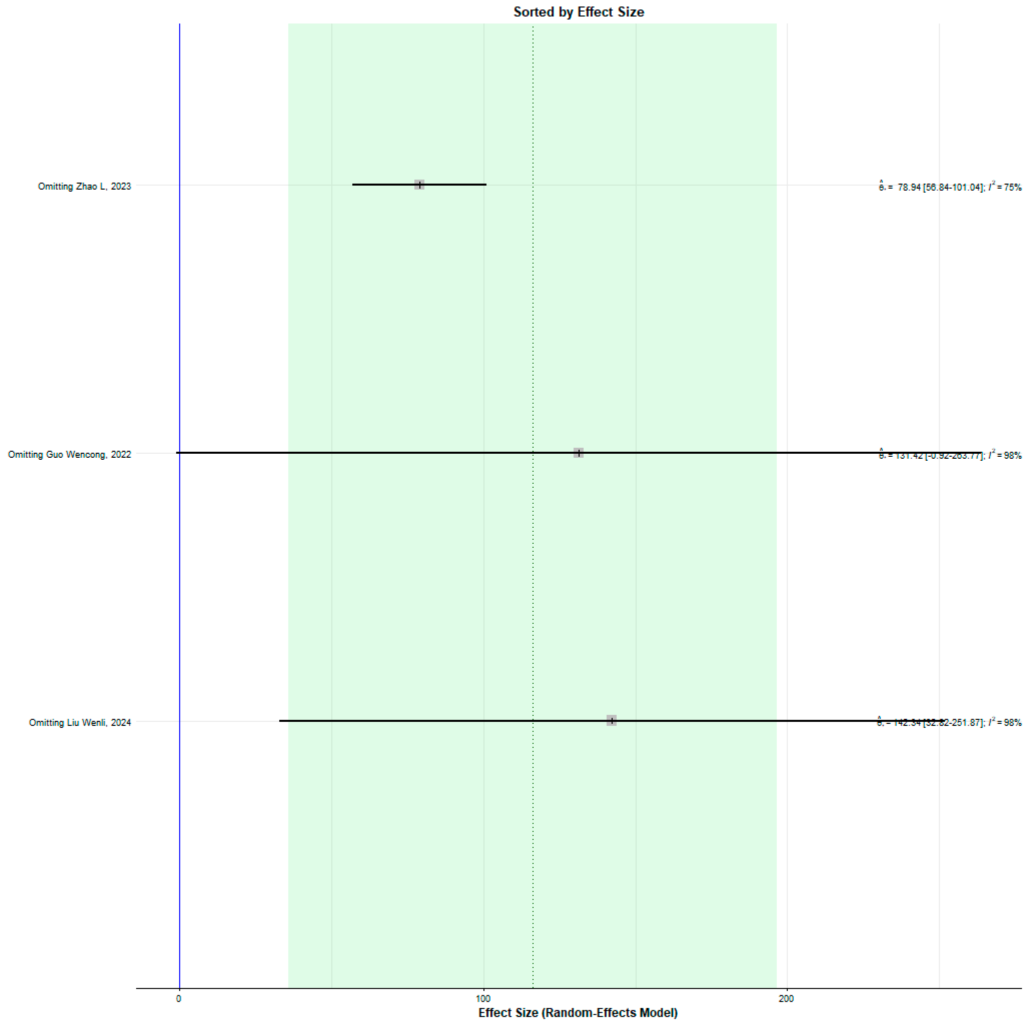

Supplementary Figure S3. Leave-one-out sensitivity analysis plot for selected studies for SII mean difference between normoalbuminuria and macroalbuminuria + microalbuminuria.

RDW mean difference between normoalbuminuria and macroalbuminuria + microalbuminuria

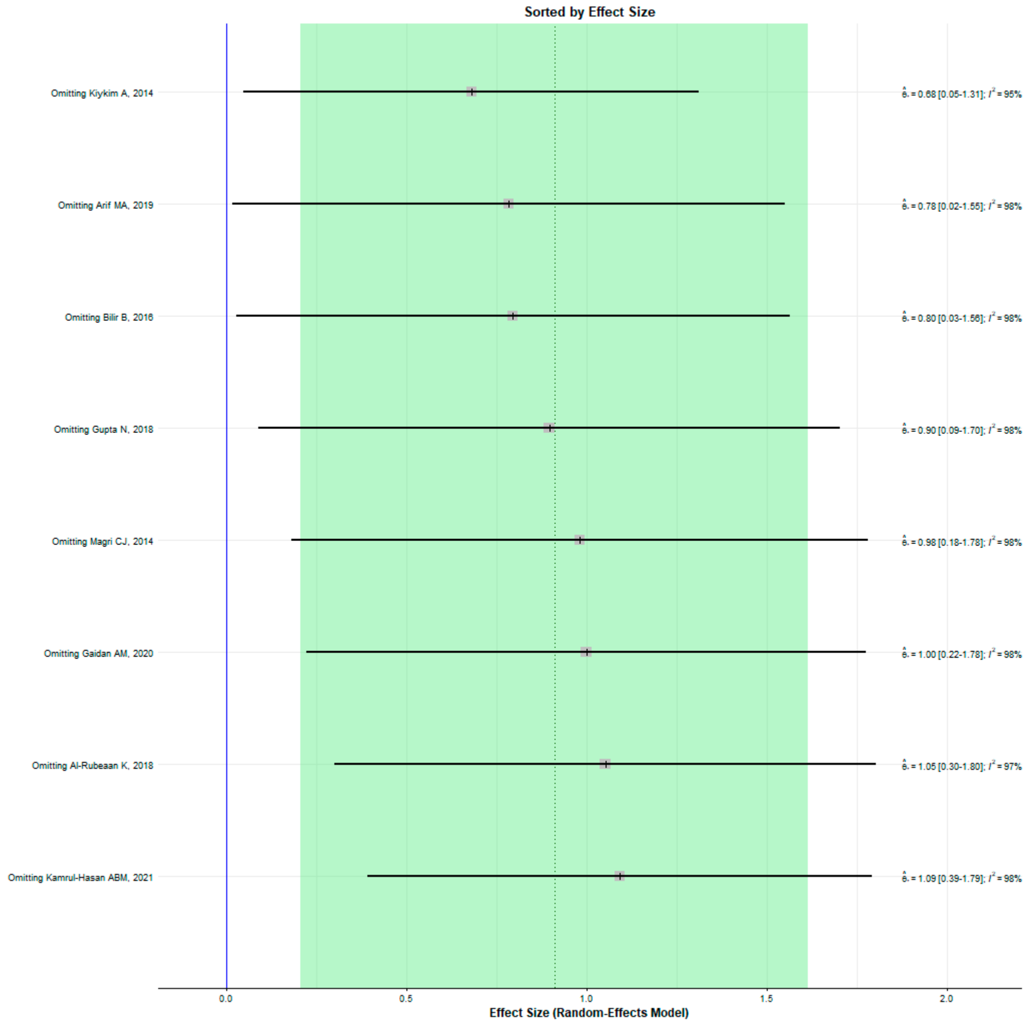

Supplementary Figure S4. Leave-one-out sensitivity analysis plot for selected studies for RDW mean difference between normoalbuminuria and macroalbuminuria + microalbuminuria.

NLR mean difference between normoalbuminuria and microalbuminuria

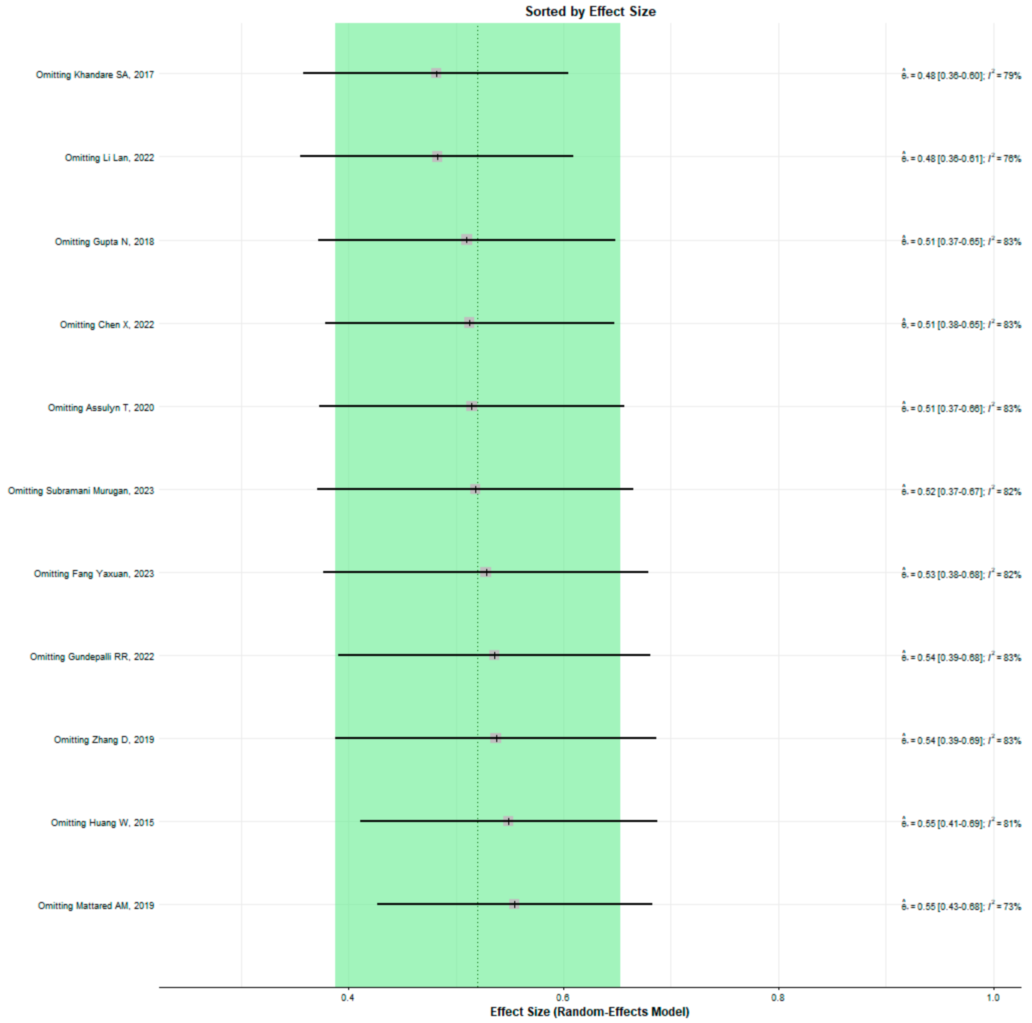

Supplementary Figure S5. Leave-one-out sensitivity analysis plot for selected studies for NLR mean difference between normoalbuminuria and microalbuminuria.

NLR odds ratio for predicting diabetic nephropathy

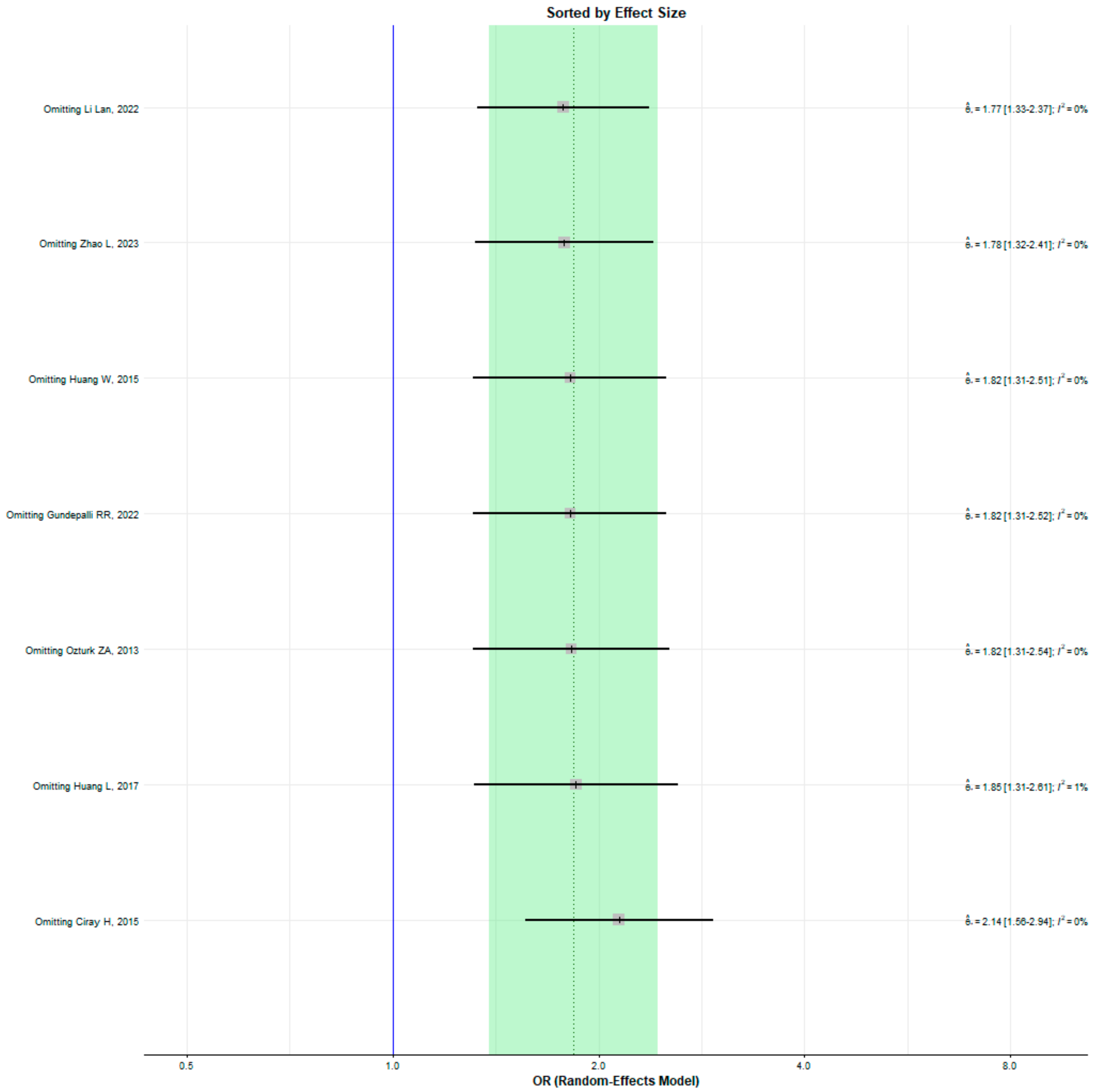

Supplementary Figure S6. Leave-one-out sensitivity analysis plot for selected studies for NLR odds ratio for predicting diabetic nephropathy.

NLR for AUC of NLR classifying between diabetic nephropathy and normoalbuminuria

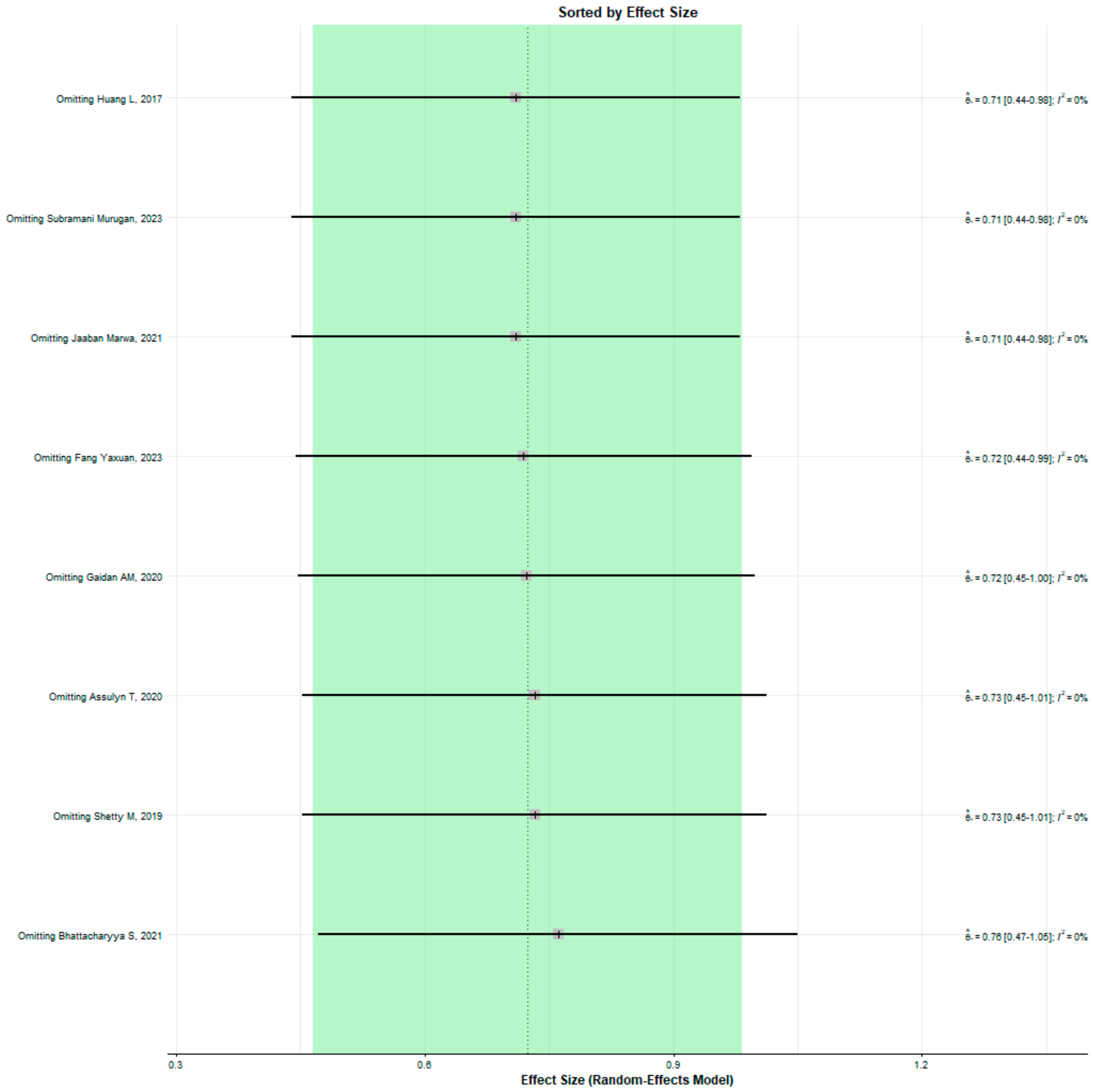

Supplementary Figure S7. Leave-one-out sensitivity analysis plot for selected studies for AUC of NLR classifying between diabetic nephropathy and normoalbuminuria.

Publication bias analyses

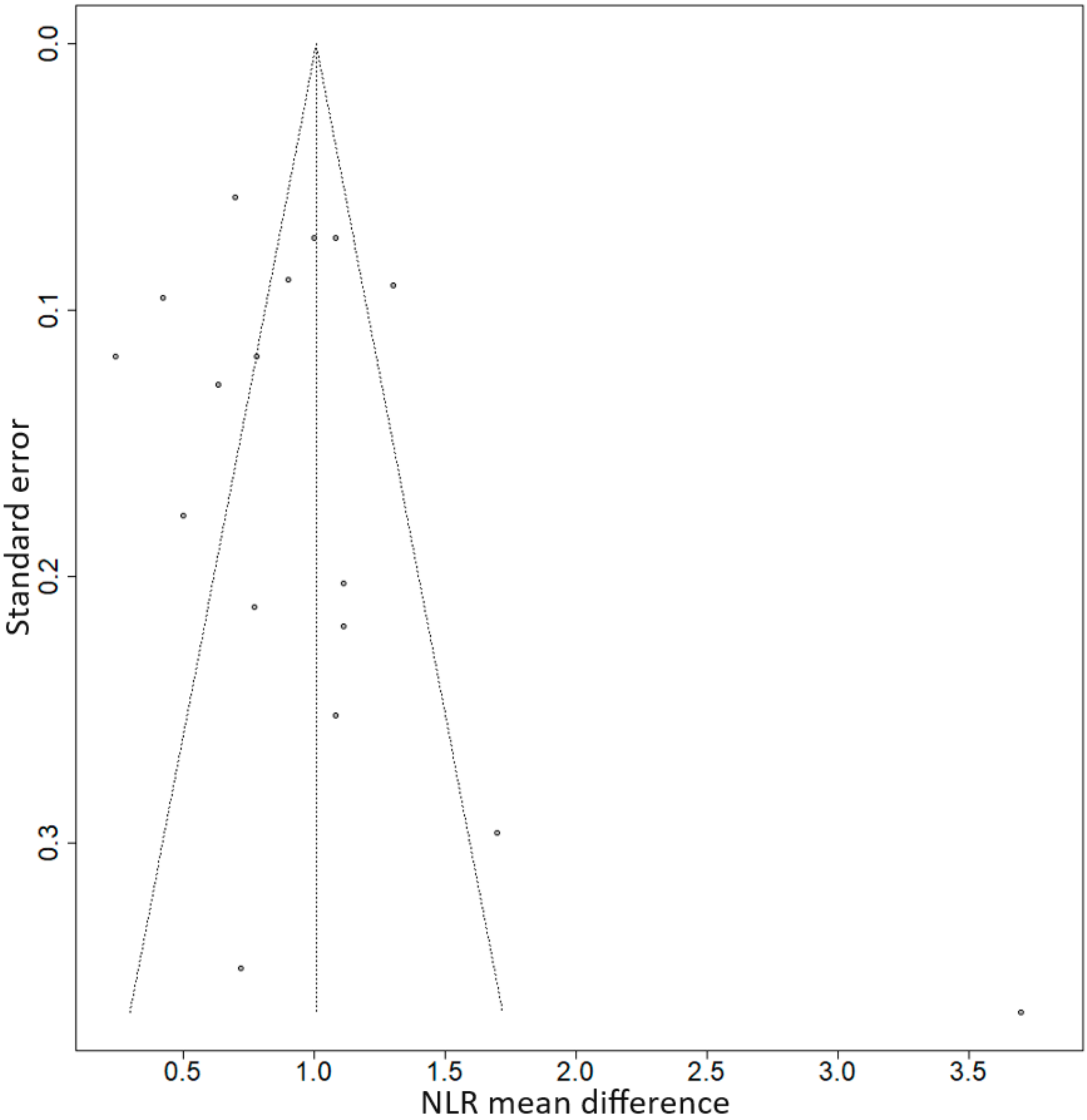

Supplementary Figure S8. **Funnel plot for the mean difference in neutrophil to lymphocyte ratio (NLR) between macro- and microabuminuria and normoalbuminuria for patients with type 2 diabetes.**

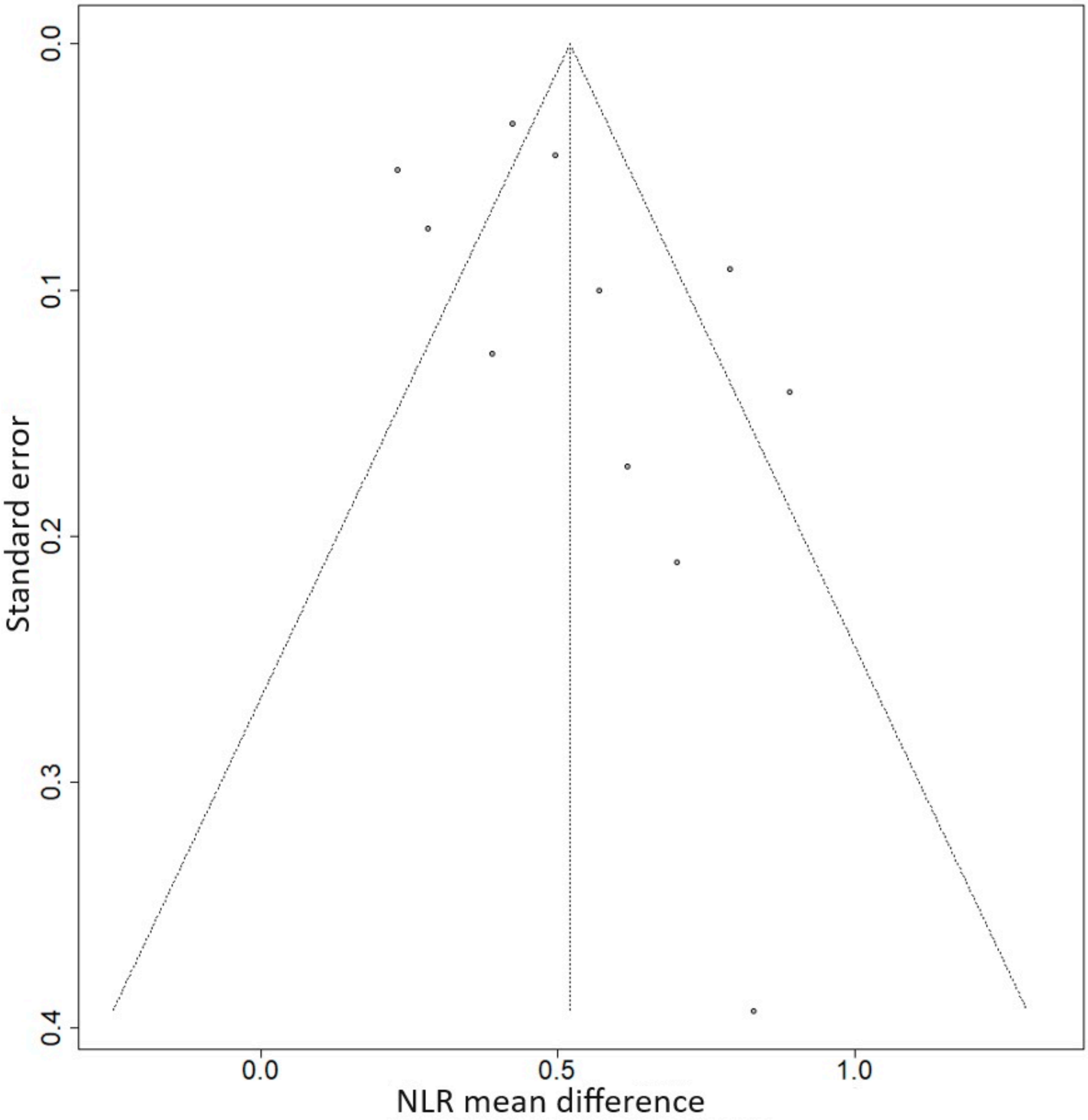

Supplementary Figure S9. **Funnel plot for the mean difference in neutrophil to lymphocyte ratio (NLR) between microabuminuria and normoalbuminuria for patients with type 2 diabetes.**

Regression results predicting diabetic nepthropathy

Supplementary Table S2. Logistic regression results predicting diabetic nephropathy for neutrophile to lymphocite ratio study results.

Study name	Number items intervention	Number items control	Cutoff?	OREPNLR	ORESNLR	ORLINLR	ORLSNLR	NLR Adjusted for	OREPNLR log	ORESNLR log
Öztürk ZA, 2013	37	205	continuous	2.035	1.160714	1.259	3.291	Gender, hypertension	0.7105	0.24512
Ciray H, 2015	62	52	continuous	1.023	1.119388	1.023	3.365	duration of diabetes mellitus	0.02274	0.30375
Huang W, 2015	115	138	continuous	2.088	1.19898	1.271	3.429	creatinine, total cholesterol, TG, systolic blood pressure (SBP), diastolic blood pressure (DBP), smoking, BMI, HbA1c and insulin resistance (IR)	0.73621	0.25318
Huang L, 2017	187	134	continuous	1.898	1.081633	1.176	3.064	Gender, Age, SBP, DBP, Hemoglobin, BMI, Smoking, Drinking, UA, Albumin, TC, LDL, HbA1c, creatinine	0.6408	0.24429
Gaidan AM, 2020	58	72	>=3.5	13.75	3.408163	1.68	11.68	duree>=5.5y, HbA1c5 >= 9, WBC *10^3/mL >=8.5	2.62104	0.49466

Li Lan, 2022	365	290	continuous	3.137	1.782653	1.955	5.033	Duration of diabetes, NLR, PLR, serum creatinine, LDL cholesterol, HbA1c	1.14327	0.24123
Gundepalli RR, 2022	69	46	continuous	2.081	1.2010 2	1.267	3.441	Creatinine, Chol Tot, TG, SBP, DBP, Smoking, BMI, HbA1c, insulin resistance	0.73285	0.25488
Zhao L, 2023	215	112	continuous	2.565	1.608418	1.32	4.985	T2DM disease course, hypertension history, FPG, LDL, BUN, Scr, UACR, neutrophil count, platelet count, NLR, PLR, SII	0.94196	0.33898
Fang Yaxuan, 2023	41	30	probable cutoff	6.562	5.857653	2.06	20.902	hs-CRP, MCP-1	1.8813	0.59111

NLR, neutrophil to lymphocite ratio; OR, odds ratio; EP, point estimator; ES, standard error; LL, lower limit of the 95% confidence interval; UL, upper limit of the 95% confidence interval; log, natural logarithm.

Supplementary Table S3. Logistic regression results predicting diabetic nephropathy for platelets to lymphocite ratio study results.

**Study name**	**Cutoff PLR**	**OR EP PLR**	**OR ES PLR**	**OR LL PLR**	**OR UL PLR**	**PLR Adjusted for**	**OR EP PLR log**	**OR ES PLR log**
Li Lan, 2022	continuous	1.008	0.514031	1	1.015	Duration of diabetes, NLR, PLR, serum creatinine, LDL cholesterol, HbA1c	0.00797	0.0038
Zhao L, 2023	continuous	0.984	0.502296	0.968	1.001	T2DM disease course, hypertension history, FPG, LDL, BUN, Scr, UACR, neutrophil count, platelet count, NLR, PLR, SII	-0.0161	0.00855

PLR, platelets to lymphocite ratio; OR, odds ratio; EP, point estimator; ES, standard error; LL, lower limit of the 95% confidence interval; UL, upper limit of the 95% confidence interval; log, natural logarithm.

Supplementary Table S4. Logistic regression results predicting diabetic nephropathy for systemic inflammation index study results.

Study name	Cutoff SII	OREPSII	ORESSII	ORLLSII	ORULSII	SII Adjusted for	OREPSII log	ORESSII log
Guo Wencong, 2022	≥445.21	1.42		1.1	1.83	age, gender, poverty income ratio, race, education levels, smoking status, Hypertension, Hyperlipidemia, Metabolic syndrome, anti-inflammation therapy, metformin use, insulin use, BMI, SBP, DBP, glycohemoglobin, serum creatinine, blood urea nitrogen, serum uric acid, eGFR, TC, TG, ALT, AST, ASCVD, and CHF.	0.35066	0.12985
Zhao L, 2023	continuous	1.011	0.515816	1.007	1.015	T2DM disease course, hypertension history, FPG, LDL, BUN, Scr, UACR, neutrophil count, platelet count, NLR, PLR, SII	0.01094	0.00202
Liu Wenli, 2024	continuous	1.001	0.510714	1	1.002	none	0.001	0.00051

SII, systemic inflammation index; OR, odds ratio; EP, point estimator; ES, standard error; LL, lower limit of the 95% confidence interval; UL, upper limit of the 95% confidence interval; log, natural logarithm.

Supplementary Table S5. Logistic regression results predicting diabetic nephropathy for red cell distribution widht study results.

Study name	Cutoff RDW	OR EP RDW	OR ES RDW	OR LL RDW	OR UL RDW	Adjusted for	OR EP RDW log	OR ES RDW log
Magri CJ, 2014	continuous	1.64	0.892857	1.15	2.35	eGFR, Night-time diastolic BP, ESR	0.4947	0.18231
Zhang M, 2015	continuous	3.89	2.459184	1.98	7.66	smoking, waist index, FBG, uric acid	1.35841	0.34513
Al-Rubeaan K, 2018	continuous	0.72	0.44898	0.38	1.38	age, diabetes duration, BMI, SBP and DBP	-0.3285	0.329

RDW, red cell distribution width; OR, odds ratio; EP, point estimator; ES, standard error; LL, lower limit of the 95% confidence interval; UL, upper limit of the 95% confidence interval; log, natural logarithm.

Supplementary Table S6. Cox regression results predicting diabetic nephropathy for SII study results.

Study name	HR EP	HR ES	HR CI LI	HR CI LS	Adjusted for
Liu Wenli, 2024	3.24	2.572449	1.179	8.905	age, sex, BMI, hypertension, duration of diabetes, and

					diabetic retinopathy

HR, odds ratio; EP, point estimator; ES, standard error; LL, lower limit of the 95% confidence interval; UL, upper limit of the 95% confidence interval

Area under the curve study results

Area under the curve for NLR study results

Supplementary Table S7. Area under the curve for neutrophile to lymphocite ratio study results.

**Study name**	**Comparison**	**Number items intervention**	**Number items control**	**AUC EP NLR**	**AUC ES NLR**	**AUC LL NLR**	**AUC UL NLR**	**Cutoff NLR**	**Se NLR**	**Sp NLR**
Öztürk ZA, 2013	macro, micro vs. normo	37	205	0.538	NA			2.92	48	89
Huang L, 2017	macro, micro vs. normo	187	134	0.872	0.445153	0.834	0.911	1.758	75.4	92.5
Shetty M, 2019	macro, micro vs. normo	156	40	0.673	0.343367	0.597	0.749	2.752	74	50
Mattared AM, 2019	micro vs. normo	30	30	0.98	NA				96	91
Assulyn T, 2020	micro vs. normo	58	110	0.675	0.341837	0.58	0.76	2.54	39.7	78.8
Gaidan AM, 2020	macro, micro vs. normo	58	72	0.736	0.380102	0.653	0.837	3.35	69	89
Bhattacharyya S, 2021	macro, micro vs. normo	39	41	0.578	0.293112	0.462	0.687	3.65	69.24	77.61

Jaaban Marwa, 2021	macro, micro vs. normo	91	67	0.869	0.443622	0.813	0.926	2.2 predicting microalbuminuria	72	78
Chen X, 2022	micro vs. normo	144	49	0.751	NA			1.56	90.24	45.9
Li Lan, 2022	macro, micro vs. normo	365	290	0.794				2.46	0.762	0.707
Singh A, 2022	macro vs. normo	146	178	0.882	NA			3.28	89.7	69.7
Moh Mei Chung, 2023	macro, micro vs. normo			0.56				1.9	0.5	0.62
Subramani Murugan, 2023	micro vs. normo	50	67	0.869	0.443622	0.813	0.926	2.2	72.3	78.1
Fang Yaxuan, 2023	micro vs. normo	41	30	0.76	0.387755	0.657	0.863	2.239	58.3	90

Area under the curve for PLR study results

Supplementary Table S8. Area under the curve for platelets to lymphocite ratio study results.

**Study name**	**Comparison**	**Number items intervention**	**Number items control**	**AUC EP PLR**	**AUC ES PLR**	**AUC LL PLR**	**AUC UL PLR**	**Cutoff PLR**	**Se PLR**	**Sp PLR**
Zahid Kocak M, 2018	macro, micro vs. normo	76	86	0.581	NA			112	60.5	54.7
Jaaban Marwa, 2021	macro, micro vs. normo	91	67	0.739	0.376786	0.662	0.815	115.6 predicting microalbuminuria	78.1	88

Chen X, 2022	micro vs. normo	144	49	0.922	NA			68.86	83.7	82.6
Li Lan, 2022	macro, micro vs. normo	365	290		0.665			125.04	0.515	0.755
Tan J, 2023	micro vs. normo	55	55	0.82					70.83	86.34

Supplementary Table S9. Area under the curve for red cell distribution widht study results.

Study file func	Study name	Number items intervention	Number items control	AUC EP RDW	AUC ES RDW	AUC LL RDW	AUC UL RDW	Cutoff RDW	Se RDW	Sp RDW
203_Zhang, M., 2015	Zhang M, 2015	118	202	0.79	0.403061	0.74	0.84	12.8	71.3	66.9
127_Assulyn, T., 2020	Assulyn T, 2020	58	110	0.614	0.311224	0.52	0.7	78.8	37.9	76
